# A delayed diagnosis of late-onset pulmonary hemorrhage in a toddler with Henoch-Schönlein purpura after regression of skin rash: A case report and literature review

**DOI:** 10.1097/MD.0000000000023025

**Published:** 2020-10-30

**Authors:** Hongyu Duan, Yimin Hua, Kaiyu Zhou, Yifei Li, Chuan Wang

**Affiliations:** a Department of Pediatrics; b The Cardiac development and early intervention unit, West China Institute of Women and Children's Health, West China Second University Hospital, Sichuan University, Chengdu; c Key Laboratory of Birth Defects and Related Diseases of Women and Children (Sichuan University), Ministry of Education Chengdu, Sichuan, China.

**Keywords:** children, delayed diagnosis, Henoch-Schönlein purpura, late-onset, pulmonary hemorrhage

## Abstract

**Rationale:**

Pulmonary hemorrhage is a rare but fatal complication of Henoch-Schönlein purpura (HSP), and more easily ignored in children than in adults due to the absence of clinically evident hemoptysis. Moreover, despite being sporadically reported, given that pulmonary hemorrhage may develop after regression and even disappearance of skin rash, the asynchronous progression of skin and lung lesions poses escalating challenges in the timely diagnosis. We herein presented a delayed diagnosis of late-onset pulmonary hemorrhage in a child with HSP after regression of purpuric rash.

**Patient concerns:**

A 6-year and 3-month child with a history of self-resolved purpuric rash three weeks ago, presented acutely with cough and dyspnea but without fever.

**Diagnoses:**

The decreased hemoglobin and diffuse ground-glass opacities of both lungs on CT scan weren’t comprehensively evaluated. The child was initially misdiagnosed as pneumonia.

**Interventions:**

Antibiotic treatment was initiated. However, no improvement of respiratory status was found following aggressive combination therapy. Bronchoscopy was subsequently performed.

**Outcomes:**

An diffuse alveolar hemorrhage with low inflammatory profile was noted after a bronchoscopy. Considering the history of HSP, the diagnosis of HSP-associated pulmonary hemorrhage was ultimately confirmed and the patient received corticosteroids with satisfactory results.

**Lessons:**

Pulmonary hemorrhage could occur in children with HSP at late onset of disease after regression of skin rash. New-onset respiratory symptoms in patients with a history of HSP should heighten suspicion for pulmonary hemorrhage, particularly if presenting with lack of fever, sudden drop of hemoglobin, new pulmonary infiltrates and unresponsiveness to antibiotics therapy. Bronchoscopy should be performed early to confirm the diagnosis, specifically for children.

## 1 Introduction

Henoch-Schönlein purpura (HSP) is a widespread leukocytoclastic vasculitis characterized by immunoglobulin A (IgA) deposition into the small vessels of multiple organ systems.^[1]^ A palpable purpuric rash is the predominant and essential sign for diagnosis. Abdominal pain and arthritis/arthralgia also usually occur in approximately 80% of the cases. Renal involvement, mainly manifesting as glomerulonephritis, nephrotic syndrome, and acute renal failure, is a well-recognized and common complication of HSP, affecting 20% to 50% of the children.^[2]^

In contrast, pulmonary hemorrhage is an extremely rare complication of HSP ranged from 0.8% to 5%, which has been mainly observed in adults.^[3,4]^ Without timely diagnosis and targeted therapies, it is always associated with significant morbidity and mortality (persistent urinary abnormalities, chronic renal failure, complications of therapy).^[5]^ Although pulmonary hemorrhage commonly occurs in the early stage of HSP, it could also onset late even after regression and disappearance of skin rash, which probably divert the diagnosis toward other respiratory diseases and pose great challenges in the early and timely detection. Furthermore, pulmonary hemorrhage is more likely to be ignored in children than adults due to the absence of clinically evident hemoptysis.^[6–11]^ In China, due to the limited recognition and experience of pediatricians, a delayed or missed diagnosis of pulmonary hemorrhage in HSP children is always more common and concerning. We herein presented a delayed diagnosis of late-onset pulmonary hemorrhage in a 6-year and 3-month child with HSP and have given a literature review, aiming to share some experience and improve the recognition of this rare complication in children with HSP for the pediatricians, particularly those in developing countries.

## 2 Case report

A 6-year and 3-month male child was referred to our hospital due to 5 days of cough. No fever and hemoptysis were reported. About 3 weeks prior, he had a 5-day history of erythematous rash dominantly involving his lower extremities bilaterally, without symptoms of arthralgia, hematochezia, or abdominal pain. Rash resolved spontaneously with no intervention. He had no history of previous disease or drug use. His family history was unremarkable. On physical examination, the patient was clear, and afebrile. He was pale in appearance without jaundice. The vital signs were: blood pressure of 93/58 mm Hg, heart rate of 106 beats/minute, respiratory rate of 24 breaths/minute, body temperature of 36.5°C, oxygen saturation of 96% (on air). He had normal respiratory effort. Lung auscultation showed crackles in bases bilaterally. There was obsolete palpable purpura over the lower limbs, no fading when compressed and symmetric in distribution. The rest of the physical examination was unremarkable.

Laboratory evaluation revealed a slight decrease in hemoglobin level (9.9 g/dl) with normal white blood cells. C-reactive protein (CRP) was 1.2 mg/dl (normal<0.8 mg/dl). Platelets, prothrombin time, partial thromboplastin time, and international normalized ratio were all within normal limits. Urinalysis, stool occult blood, serum electrolytes, blood gas analysis, liver function test, and creatinine level were unremarkable. A chest radiography indicated bibasilar airspace opacities suggestive of multifocal pneumonia. Consequently, intravenous cefathiamidine combined with mezlocillin were started empirically.

**Figure 1:**
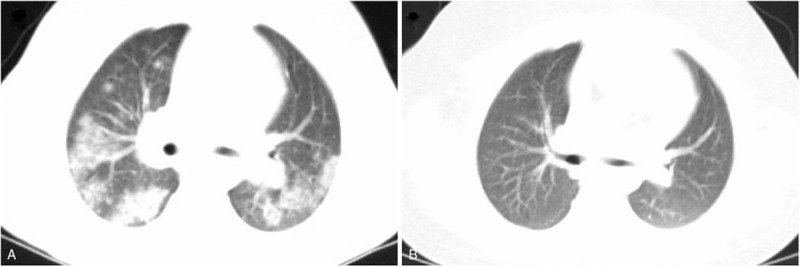
Pulmonary CT images prior to and following treatment. Diffuse patchy ground-glass opacities were present predominantly on the inferior lobe dorsal segments of the both lungs without interstitial thickening and fibrosis (A). Following treatment, the original pulmonary hemorrhage was clearly absorbed and reduced (B).

**Figure 2:**
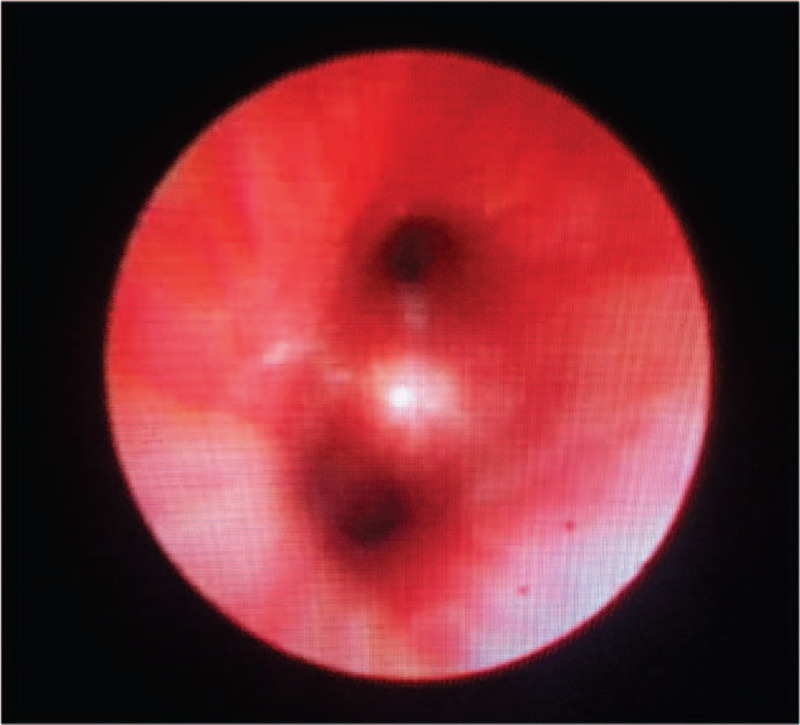
Fiber-optic bronchoscopy showed diffuse mucosal heperemia and edema accompanied by derangement and varices of submucosal vessels in bronchial trees.

On day 3 of admission, the patient began grunting, had respiratory rates of 42 to 50 breaths/minute, and a blood gas revealed a pH of 7.45 and pCO_2_ 30 mm Hg with 27% oxygen inhalation via a nasal cannula. Results of sputum culture, chlamydia/ mycoplasma antibody, PPD test, T-spot assay, serum glucan/galactomannan tests, and blood culture were negative. Repeat hemoglobin and CRP were 8.2 g/dl and 2.0 mg/dl, respectively. To further investigate the nature of the pulmonary lesion, an enhanced chest computed tomography (CT) was conducted that demonstrated diffuse ground-glass opacities of the inferior lobes of both lungs with pleural thickening (Fig. 1A), but the property was unclear. Antibiotics were switched to vancomycin, ceftriaxone, and azithromycin for presumed severe infection. Unfortunately, the effects seemed discouraging as well, and no improvement of respiratory status was found following 3 days of treatment. Considering the frustrating effect of antibiotic therapy, we had to refer to our pediatric pulmonologist. After discussion and clinic consultation, for further evaluating the underlying cause and severity of the airway lesions, bronchoscopy was arranged, so that an appropriate treatment regimen was able to be adopted. Bronchoscopy revealed normal bronchial pathway and absence of acute hemorrhage, but with diffuse mucosal heperemia and edema accompanied by derangement and varices of submucosal vessels in bronchial trees (Fig. 2). Bronchoalveolar lavage fluid was noted to be bloody. Further cytological analysis demonstrated low inflammatory profile (cell differential: 52% macrophages, 47% neutrophils, <1% lymphocytes, and <1% eosinophils), and numerous hemosiderin-laden macrophages. Thus, a diagnosis of diffuse alveolar hemorrhage (DAH) was confirmed. Results of lavage specimen for fungal culture, respiratory culture, acid-fast stain, and viral polymerase chain reaction were all negative. Antibiotics were weaned in view of negative microbiological workup. However, the specific etiology of DAH needed to be identified. Although pulmonary hemorrhage of our case occurred late after exacerbations of skin rash, we also assumed that DAH was more likely related to HSP because of considering the characteristic cutaneous lesions. For further differential diagnosis of DAH in children, anti-glomerular basal membrane antibodies (anti-GBM), antinuclear antibody (ANA), anti-neutrophil cytoplasmic antibody (ANCA), and complement (C3 and C4) were performed, all the results being negative. Ultimately, on account of a palpable purpuric rash, worsening anemia, poor response to antibiotic therapy, and the results of bronchoscopy, HSP-associated pulmonary hemorrhage was confirmed.

The patient was then given pulse methylprednisolone intravenously (30 mg kg^−1^·d^−1^ for 3 consecutive days) followed by oral prednisone (2 mg kg^−1^·d^−1^). After 6 days of treatment, he symptomatically improved with complete resolution of cough, tachypnea, and purpura. A repeat chest CT demonstrated that original opacities of the lung fields were markedly reduced (Fig. 1B). The patient was discharged and continued prednisone. Since the patient was asymptomatic on monthly follow-up visits, the steroid dose was gradually tapered, and discontinued after 6 months.

## 3 Discussion

Pulmonary hemorrhage is a rare but frequently fatal complication of HSP.^[4,12,13]^ For pediatric patients, we performed a research of the literature describing HSP with pulmonary hemorrhage in PUBMED Database. Totally, since 1979, 24 pediatric cases were identified. The main findings of these cases were shown in Table 1. The average age of pulmonary hemorrhage in HSP was 10.4 years (range, 1.75–18 years), affecting 13 males and 11 females (M/F ratio = 1.18). The median duration between onset of HSP and pulmonary hemorrhage was 11 days, ranging from 2 days to 28 weeks,^[4,6–11,14–26]^ with the exception of 2 cases in which the time was not reported due to absence of the relevant symptoms.^[15,23]^ All the patients had renal involvement, ranging from hematuria commonly accompanied with proteinuria, to nephritis through to acute renal failure, some of whom even showing synchronous progression with lung lesions.^[4,6–11,14–26]^ Additionally, One third of the patients had other organ involvement, notably neurologic (16.7%) or cardiac system (16.7%), contributing to high mortality and morbidity.^[4,7,8,10,14,22,25]^ Moreover, thirteen children (54.2%) required intubation and mechanical ventilation due to respiratory distress and massive pulmonary hemorrhage.^[4,8–11,17–20,22,24,25]^ To our knowledge, our case was the first one of HSP with late-onset pulmonary hemorrhage after regression of skin rash and no renal involvement in children, which significantly contributed to our delayed diagnosis. In spite of the benign prognosis in our case, early diagnosis and prompt management are clinically substantial since most of HSP cases with pulmonary hemorrhage present with a poor prognosis.

**Table 1:**
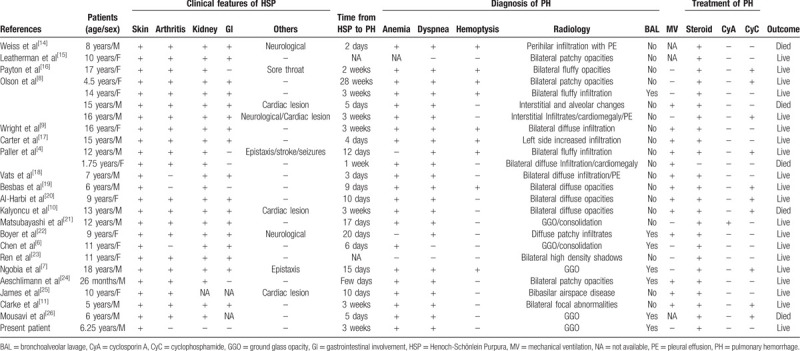
Summary of all reported cases of pediatric patients with pulmonary hemorrhage and Henoch-Schönlein purpura in English literature.

However, a variety of factors may divert the diagnosis toward other respiratory diseases, resulting in a delayed diagnosis of pulmonary hemorrhage. First, although most of patients show concurrent purpura at onset of pulmonary hemorrhage, skin involvement may occur variably, or some cases even exhibited no purpura throughout the entire clinical course.^[5,7]^ Overall, the time of lung involvement ranged from 2 days to 18 years (mean time: 3 weeks) after diagnosis of HSP.^[5]^ Of note, it seemed that onset of pulmonary hemorrhage during the course of the disease tended to occur much earlier in children and young adults than those with advanced or very young age.^[4,7,21,24,25]^ As the occurrence of pulmonary hemorrhage is immune-mediated, it is therefore possible that immune status related to the age is likely a contributor to the time of lung involvement. Meanwhile, the development of pulmonary hemorrhage is not in good accordance with exacerbations of skin rash, which may occur during or after the exacerbations.^[23]^ Therefore, the asynchronous progression of skin and lung lesions described above poses challenges in the detection of pulmonary hemorrhage. Additionally, the clinical manifestations of pulmonary hemorrhage in HSP vary significantly, ranging from mild cough to severe tachypnea and dyspnea, even respiratory failure. Clinically evident hemoptysis is absent in almost two-third of patients.^[4,6,8,10,11,15,18,20–26]^ Over 10% of the patients in children even had no respiratory symptoms, but only radiological imaging findings suggestive of pulmonary hemorrhage.^[6,15,23]^ Moreover, imaging findings of patients with pulmonary hemorrhage are variable and nonspecific, including mild perihilar infiltrates, and unilateral or bilateral ground-glass opacities with pleural involvement.^[5–7,14,21,26]^ These varied findings only permit a general diagnosis of pulmonary involvement without favoring a specific etiology.

As for our case, the cough and acute dyspnea developed late in the course of the disease (up to 3 weeks from the onset of purpura) and progressed after improvement of the purpura, resulting in ignorance about the correlation between the respiratory symptoms and previous rash, a significant clue to the diagnosis. Additionally, although renal involvement was reported as the most common associated symptoms at the onset or progression of pulmonary hemorrhage,^[6,7,11,22–24]^ no corresponding findings were identified in our case, resulting in delayed recognition of disease association with HSP. Additionally, in spite of a drop in hemoglobin level and lack of fever, the property of diffuse infiltrates in both of lung areas was initially considered as infection due to the limited recognition of pulmonary hemorrhage. Regrettably, despite poor response to antibiotic treatment and negative findings of microorganism, pulmonary hemorrhage was still not suspected in the differential diagnosis until after a bronchoscopy had been performed.

On account of a review of the literature and experience of us, early and prompt recognition may be achieved in the following manners. First, pulmonary hemorrhage should always be considered in the differential diagnosis in patients with history of HSP in case of respiratory symptoms. Second, lack of fever, sudden drop of hemoglobin without gross hematuria and bloody stools, new diffuse pulmonary infiltrates might heighten suspicion for pulmonary hemorrhage in patients with history of HSP,^[7,24]^ particularly those refractory to antibiotic treatment. Finally, bronchoscopy with bronchoalveolar lavage should be performed early in suspect cases to facilitate confirmation of diagnosis and exclusion of possible infections, even if hemoptysis is not observed.^[6,8,22,24,26]^

Although standard treatment for HSP-associated pulmonary hemorrhage has not been established, most authors recommended intravenous pulse methylprednisolone, followed by oral steroids.^[27]^ The addition of immunosuppressive agents (i.e., cyclophosphamide and cyclosporin A) to corticosteroids for the treatment strategies remains controversial. Optimal treatment strategies might be preferably based on severity of lung or other organ system involvement. Combined therapy might be indicated in children with severe organ involvement, commonly respiratory failure and crescentic glomerulonephritis, in order to achieve better outcomes.^[6,21]^ Fortunately, our case had a favorable outcome with corticosteroids, which was probably attributed to absence of respiratory failure and clinically evident renal involvement. Considering the significant adverse effects of immunosuppressant therapy, it should be used prudently based on sound clinical judgment. Additionally, limited data are available for the prevention and treatment of lung involvement in HSP, especially in pediatric patients. Further multicentric studies with a standard protocol are required to define the optimal therapeutic strategies in patients, particularly pediatric patients, with pulmonary hemorrhage related to HSP.

## 4 Conclusions

Pulmonary hemorrhage is a rare complication of HSP in children and it could onset late after regression and disappearance of skin rash. The asynchronous progression of skin and lung lesions, and absence of clinically evident hemoptysis in children pose great challenges in the early and timely detection of pulmonary hemorrhage. New-onset respiratory symptoms in patients with a history of HSP should heighten suspicion for pulmonary hemorrhage, particularly if presenting with lack of fever, sudden drop of hemoglobin, new pulmonary infiltrates and unresponsiveness to antibiotics therapy. Bronchoscopy with bronchoalveolar lavage should be performed early to confirm the diagnosis, particularly in children.

## Acknowledgments

We thank the funding supporters [National Natural Science Foundation of China (No. 81971457, No. 81602817 and No. 81800288), and Science-technology Plan Projects of Applied Basic Research in Sichuan province (2020YJ0234)].

## Author contributions

**Conceptualization:** Hongyu Duan, Yimin Hua, Kaiyu Zhou.

**Data curation:** Hongyu Duan, Yimin Hua, Kaiyu Zhou.

**Methodology:** Hongyu Duan.

**Resources:** Hongyu Duan, Yimin Hua, Kaiyu Zhou.

**Supervision:** Chuan Wang.

**Writing – original draft:** Hongyu Duan.

**Writing – review & editing:** Yifei Li, Chuan Wang.
